# Quality of hip and knee osteoarthritis management in primary health care in a Norwegian county: a cross-sectional survey

**DOI:** 10.1186/s12913-014-0598-x

**Published:** 2014-11-25

**Authors:** Gudmund Grønhaug, Nina Østerås, Kåre Birger Hagen

**Affiliations:** National Resource Centre for Rehabilitation in Rheumatology, Department of Rheumatology, Diakonhjemmet Hospital, P.O. Box 23 Vinderen, 0319 Oslo, Norway

**Keywords:** Osteoarthritis, Quality indicator, Primary care, General practitioner, Knee, Hip

## Abstract

**Background:**

Osteoarthritis (OA) is one of the most common causes of pain and disability in the adult population. Several studies have documented discordance between general practioners (GP) practice and management recommendations, but there is limited published information about patient reported experience of quality of care. The primary aim of this study was to assess the patient perceived quality of OA management in primary health care. Secondly, we wanted to explore the factors associated with the perceived quality of OA care.

**Methods:**

A cross-sectional survey in six general practices in the county of Nord-Trøndelag in Norway, patients with radiologically diagnosed OA, according to ICPC codes L89, L90 or L91 or clinical signs and symptoms corresponding to OA in the hip or knee and patient-reported quality of OA care on the 17-item OsteoArthritis Quality Indicator questionnaire (OA-QI). OA-QI summary pass rates were calculated, in which the numerator represents the number with indicators passed and the denominator represents the total number of eligible persons. Associations with summary pass rates were explored with demographic, disease related and health care related factors as independent variables.

**Results:**

A total of 119 patients were included (response rate 42%). The median summary QI pass rate for all 17 QIs was 47% (Inter Quartile Range 33-65%), but there were large variation between the different items. The referral for weight reduction had the lowest pass rate (8%), whereas the highest pass rate was having received information about the importance of physical activity and exercise (84%). The median summary QI pass rates for both non-pharmacological- (QIs 1–11) and pharmacological (QIs 13–16) treatments were 50% (IQR 25–75). In bivariate regression analyses, only overall treatment satisfaction was significantly associated with QI pass rate (p = 0.001), with unstandardized beta = 6.1 (95% CI 2.7 to 9.5), i.e. a one-point increase on the five-point satisfaction scale was associated with a 6% increase in pass rate.

**Conclusion:**

Considering that the median summary QI pass rate was 47%, there might be room for improvement in OA care. Advice and the referral of OA patients in need of weight reduction seem to have the greatest potential for improvement.

**Electronic supplementary material:**

The online version of this article (doi:10.1186/s12913-014-0598-x) contains supplementary material, which is available to authorized users.

## Background

Osteoarthritis (OA) is one of the most common causes of pain and disability in the adult population [1]. The prevalence of OA is related to age and body weight, and due to an increase in age and body weight in the population, the prevalence of OA is expected to further increase. Furthermore, it is estimated that 15-25% of visits to the general practitioner related to musculoskeletal conditions are due to OA [2]. Evidence-based guidelines have been consistent for more than a decade [3-6], advising patients to make use of core treatments such as exercise and weight reduction. Transferring guidelines into practice is not easy and knowledge and adherence to guidelines in daily practice is limited [7,8], thereby resulting in suboptimal OA treatment [9-11]. It is further estimated that OA alone results in greater costs than all other rheumatic diseases in relation to sick leave and comorbidities [12,13]. If OA patients were given evidence-based care to manage their everyday problems associated with OA, it may help to contribute to reduced disability and sick leave. Hence, both the cost to society and the burden of disease for the individual may be reduced.

One way to assess the adherence to guidelines and the quality of the treatment given is to use quality indicators (QIs), the fulfilment of which can be evaluated by the patients or health-care providers or by extracting data from patient records or other sources of information such as quality registries. A small number of QI sets for OA exist [14], including Assessing Care of Vulnerable elders (ACOVE) [15] as a part of the total care given to people 65 or older, in addition to Arthritis Foundation Quality Indicators [16], which is a checklist for the general practitioner (GP). In this study we used a recently developed instrument for patient-reported quality of OA care, the OsteoArthritis Quality Indicator questionnaire (OA-QI) [17], which assesses both the pharmacological and non-pharmacological aspects of OA treatment.

The Norwegian health care system provides universal access to primary and hospital based medical care. There is also universal access to community based physiotherapy services. Traditionally, persons with OA have received treatment in both primary and secondary care, with the Norwegian Directorate of Health recently placing the main responsibility for OA management in primary health care [18]. Thus, the main aim of this study was to capture patients’ assessment of the quality of OA care in a primary health-care setting, while we also wanted to explore factors associated with the perceived quality of OA care.

## Methods

The study was designed as a cross-sectional survey, in which patients completed a questionnaire after a GP consultation.

### Study participants

The respondents were recruited at six GP offices during a consultation (two in Namsos, two in Verdal and two in Levanger, all of which are situated in Nord-Trondelag County in the middle of Norway), with the inclusion period lasting from 10 January 2012 until 1 October 2012. The county of Nord-Trøndelag is considered to reflect the national characteristics regarding population demographics. The area where the study was obtained is comprised of minor cities and their rural surroundings, reflecting the variety of the population’s socio-economic and sociocultural background.

Patients with either radiologically diagnosed OA, according to ICPC codes L89 (osteoarthritis of the hip), L90 (osteoarthritis of knee) or L91 (osteoarthritis, other) or clinical signs and symptoms corresponding to OA in the hip or knee were considered eligible, while patients with inflammatory rheumatic diseases, malignant illness or other conditions considered by the GP to affect the persons’ abilities to complete the questionnaire were excluded. The ICPC codes are used in primary care in most countries in Western Europe. The ICPC coding is based on conceptual constructs, and is focused on health problems, as compared to the ICD framework which is based on historical constructs and is focused on disease.

### Data collection

During a consultation, the collaborating GPs handed out an envelope to all eligible patients that contained information on the study, a questionnaire and a prepaid envelope for returning the questionnaire. Both the doctors and the secretarial staff at the GP offices were involved. The medical secretaries were asked to remind eligible patients about the study after the consultation with the GP and to hand out questionnaires to those who had not been included by the GP. The offices were monitored with telephone follow-ups and personal visits by one of the researchers (GG). The personal visits were organized as lunch meetings where the recruitment procedures and number of included patients were discussed. The process of handing out questionnaires, including where to fill them in and how to return them, was not standardized due to differences in patient consultation routines, the reasons for visiting the GP, the time schedule of the treatment and how they organized their transportation. Although some respondents completed the questionnaire in the GP’s waiting room and returned them there, most brought them home and returned the envelopes by post.

### Variables

The questionnaire covered areas related to the respondents’ demographic characteristics, symptoms and health status use of health-care services and quality of OA treatment.

#### Demographic and lifestyle variables

Respondents were asked about age, height/weight, sex, occupational status (full time, part time, age retired, disability pensioner, sick leave, other), education (lower secondary school, upper secondary school, university ≥ one year), smoking habits and self-reported comorbidities.

#### Location, symptoms and health related quality of life

Respondents were asked about the site of OA (knee right/left, hip right/left, hand right/left), whether OA was their most prominent health problem and for how long they had the OA diagnosis (<one year, one-three, four-six, seven-10, >11 years). Depending on the main affection of OA as decided by the GP, disease-specific, health-related quality of life was assessed using the Hip Osteoarthritis Outcome Score (HOOS) or the Knee Osteoarthritis Outcome Score (KOOS). HOOS/KOOS consists of six subscales: symptoms (KOOS: five questions, HOOS: three), stiffness (two questions), pain (KOOS: nine questions, HOOS: 10), function in daily living (ADL) (17 questions), function in sport and recreation (Sport/Rec) (KOOS; five questions, HOOS: four) and hip/knee-related quality of life (four questions). Standardized answer options were given on a five-point Likert scale (from 0 to four points), and the results were summed up for each subscale separately and presented as a normalized score (0 indicating extreme symptoms, 100 indicating no symptoms). The HOOS questionnaire is shown in Additional file 1.

#### Use of health services

Respondents were asked about the use of health-care services during the last year, including who they had visited (GP, orthopaedic surgeon and physiotherapist) and how many consultations they had during the last year using six categorical response options, as well as how satisfied they were with the OA treatment in general on a five-point scale from very unsatisfied to very satisfied.

#### Quality of OA care

We used the OsteoArthritis Quality Indicator questionnaire (OA-QI) to further investigate what type of treatment the respondents had obtained (shown in Additional file 2). The questionnaire was developed in Norway using published QIs, expert panels and patient interviews, and was tested for reliability and validity in a Norwegian OA cohort [18]. Support for content validity was confirmed by two patient research partners and two expert panels. All ten predefined hypotheses relating to construct validity were confirmed. Test-retest Kappa coefficients ranged from 0.20-0.80 and the percent of exact agreement from 62% to 90% [18]. The OA-QI assessed whether 17 different aspects of OA treatment had been obtained using three response options (yes, no, do not remember/not relevant). The questionnaire included QIs related to patient education and information, regular provider assessments, referrals and pharmacological treatment.

### Statistical analyses

Mean and standard deviation (SD) were used to describe the participants and their characteristics for continuous variables, whereas counts with percentages were used for categorical variables. Group comparisons were performed by Chi-square for categorical data and independent sample t-tests for continuous data.

QI pass rates were calculated for each QI separately (with a 95% CI) for the study sample as a whole, in which the numerator represents the number of indicators passed (those reporting “yes”) and the denominator represents the number of eligible persons (those reporting “yes” or “no”). Correspondingly, summary pass rates for each person were calculated as the total number of QIs they passed divided by the total number of QIs for which they were eligible. Summary pass rates hip- or knee OA, as well as for the total sample, were calculated. Additionally, summary pass rates for pharmacological (QIs 13–16) versus non-pharmacological (QIs 1–11) treatments were also calculated, though due to the skewed distributions of the summary scores, the percentages are presented as a median with interquartile ranges (IQRs). Missing data were ignored in analyses; therefore, the number of persons included in the analyses varies.

Lastly, we explored sources for variation in QI pass rates in bivariate regression analyses, employing the following independent variables: demographic (age, sex, education), disease related (health-related quality of life measured by the five Koos/Hoos subscores), health-care utilization (number of visits to GP/orthopaedic surgeon/physiotherapist) and overall satisfaction with care.

### Ethics

The study sample received written information about the study, and written consents were given. The study was approved by the Norwegian Regional Committee for Medical and Health Research Ethics (Ref. no. 2011/16779).

## Results

During the nine-month inclusion period, 283 questionnaires were handed out to patients by the collaborating GPs and 119 (42%) were returned. Seventy-one persons (59.7%) reported that the main affection of OA was the knee(s), while 48 (40.3%) reported hip(s) as the most affected joint.

The mean age was 65 years (SD 10), with an average self-reported BMI of 27.4 (SD 6.3) (Table 1), 73% of which were females. A large proportion of the respondents were age retired (37%), some were disability pensioners (21%) and others worked full/part time (21%). They had a relatively high level of education, with 20% having ≥ one year of university education, 18% of respondents were smokers and as many as 72% reported another chronic disease.

**Table 1:** **Participant characteristics**

	**Knee N = 71**	**Hip N = 48**	**All N = 119**
**Age,** mean (SD)	66 (10)	64(10)	65 (10)
**BMI,** mean (SD)	28 (5,3)	26.4 (7.62)	27.4 (6.3)
**Females**, n (%)	48 (68)	39 (81)	87 (73)
**Occupational status,** n (%)			
Working	13 (18)	13(27)	26(21)
Age retired	28 (39)	16(33)	44(37)
Disability pensioner	13 (18)	8(17)	21(18)
Other/missing	16 (23)	10(21)	26(22)
**Education,** n (%)			
Lower secondary school	17 (24)	6 (13)	23 (19)
Upper secondary school	34 (48)	24 (50)	58 (49)
University	20 (28)	15 (31)	35 (20)
Missing	0	3 (6)	3 (3)
**Smoking,** n (%)			
Yes	14 (20)	7 (15)	21 (18)
No	56 (79)	40 (83)	96 (81)
Missing	1 (1)	1 (2)	2 (1)
**Comorbidity,** n (%)			
Other chronic diseases than OA	50 (70)	36 (81)	86 (72)

Fifty-four percent reported OA as their most prominent health problem, while 36% reported that OA sometimes represented their most prominent health problem (Table 2). Patients with knee OA were more often bilaterally affected (56%) than those with hip OA (44%). The majority of the respondents had the OA diagnosis for four to 10 years (41%), whereas 22% had the OA diagnosis for more than 10 years. Both the knee and hip OA groups reported high levels of pain on the KOOS/HOOS pain subscale (normalized score: KOOS: 52, HOOS: 48), and although not significant, it may seem that the knee group reported more problems on the function of daily living (ADL) subscale than the hip group (normalized score 21 vs. 42).

**Table 2:** **Localization, time since osteoarthritis (OA) diagnosis, treatment satisfaction and impact of OA**

	**Knee N = 71**	**Hip N = 48**	**All N = 119**
**Localization of the osteoarthritis disease**			
Unilateral vs. bilateral (%)	44% vs. 56%	53% vs. 47%	47% vs. 53%
Unisite vs. multisite, (%)	49% vs. 51%	57% vs. 43%	52% vs. 48%
**OA as the most prominent health problem,** n (%)			
Yes	34 (48)	30(63)	64 (54)
Yes, sometimes	29 (41)	14 (29)	43 (36)
No	8 (11)	3 (6)	11 (9)
**Time since OA diagnosis,** n (%)			
<1 year	12 (17)	7 (15)	19 (16)
1-3 years	16 (23)	6 (13)	22 (19)
4-6 years	17 (24)	12 (25)	29 (24)
7-10 years	11 (15)	9 (19)	20 (17)
>10 years	14 (20)	12 (25)	26 (22)
Missing	1 (1)	2 (4)	3 (3)
**HOOS/KOOS subscores, mean (SD)**			
Pain	52 (17)	48 (18)	
Symptoms	60 (15)	48 (17)	
Function in daily living (ADL)	52 (22)	54 (19)	
Function in sport and recreation	21 (20)	42 (22)	
Hip/Knee-related quality of Life	36 (19)	44 (17)	

Health services are frequently used (Table 3), as 55% in the knee group and 65% in the hip group visited the GP one to three times last year. In the knee OA group, 32% had been to a physiotherapist more than 12 times during the last year, while this was the case for only 15% of the hip OA patients. Additionally, 31% of the knee group and 19% of the hip group had seen an orthopaedic surgeon.

**Table 3:** **Health professionals visited last 12 months because of OA**

	**1-3 visits**		**4-6 visits**		**7-9 visits**		**10-12 visits**		**> − 12 visits**		**Never**	
	**Knee**	**Hip**	**Knee**	**Hip**	**Knee**	**Hip**	**Knee**	**Hip**	**Knee**	**Hip**	**Knee**	**Hip**
General practitioner, n (%)	39 (55)	31 (65)	15 (21)	15 (31)	4 (6)	3 (6)	2 (3)	0	1 (1)	0	10 (14)	10 (21)
Orthopaedic surgeon, n (%)	17 (24)	9 (19)	4 (6)	1 (2)	1 (1)	0	0	0	1 (1)	0	48 (68)	38 (79)
Physiotherapist, n (%)	5 (7)	3 (6)	2 (3)	1 (2)	3 (4)	3 (6)	5 (7)	4 (8)	23 (32)	7 (15)	33 (46)	30 (63)

The respondents seem to be satisfied with how their OA was being treated, as 44% reported being either pleased or very pleased with their OA treatment (Table 4), with only 7% being unpleased or very unpleased with the treatment they had received.

**Table 4:** **Self-reported treatment satisfaction**

	**Knee N = 71**	**Hip N = 48**	**All N = 119**
Very pleased, n (%)	6 (8)	3 (6)	9 (8)
Pleased, n (%)	24 (34)	19 (40)	43 (36)
Neutral, n (%)	29 (41)	18 (38)	47 (39)
Unpleased, n (%)	4 (6)	0	4 (3)
Very unpleased, n (%)	2 (3)	3 (6)	5 (4)

There were large variations in pass rates for the different QI items (Table 5). The QI about referral for weight reduction had the lowest level pass rate by far, with 8% of those who self-reported being overweight having received a referral to weight control (knee 11% and hip 4%), whereas the highest pass rate was related to having received information about the importance of physical activity and exercise at 84% (83% knee OA and 85% hip OA). Being referred to a physiotherapist or others for guidance on physical activity had the second highest pass rate at 76% (79% knee OA and 72% hip OA), while four of the QIs had pass rates below 30%.

**Table 5:** **OsteoArthritis Quality Indicator Questionnaire**

	**OA-QI items**	**Knee pass rate (% yes)**	**Hip pass rate (% yes)**	**All participants (% yes)**
**1**	Disease development	59%	51%	55%
**2**	Treatment	51%	51%	51%
**3**	Self-management	42%	43%	43%
**4**	Lifestyle	33%	41%	36%
**5**	Physical activity	83%	85%	84%
**6**	Referral physical activity	79%	72%	76%
**7**	Weight reduction	40%	40%	40%
**8**	Referral weight red.	11%	4%	8%
**9**	Functional assessment	47%	32%	40%
**10**	Walking aid assessment	26%	29%	27%
**11**	Other aids assessment	18%	17%	18%
**12**	Pain assessment	72%	52%	64%
**13**	Paracetamol	73%	69%	72%
**14**	Stronger pain killers	44%	41%	43%
**15**	NSAIDS	62%	50%	56%
**16**	Cortisone	16%	22%	17%
**17**	Referral orthopaedic surgeon	41%	41%	41%
	**Mean QI pass rate**	47%	44%	45%

The mean QI pass rate for all 17 QIs was 45%, and the median score for summary QI pass rates was 47% (IQR 33-65%). The median summary QI pass rates for both non-pharmacological (QIs 1–11) and pharmacological treatments (QIs 13–16) were 50% (IQR 25–75), and there were very low levels of missing data for the individual QIs.

In the explorative regression analyses, only overall treatment satisfaction was significantly associated with QI summary pass rate (p = 0.001), with unstandardized B = 6.1 (95% CI 2.7 to 9.5), i.e. a one-point increase on the five-point satisfaction scale was associated with a 6% increase in the pass rate. In addition, the number of physiotherapy consultations over the last year was borderline significant (p = 0.061), with unstandardized B = 1.7 (95% CI −0.8 to 3.6). The other 10 independent variables were not associated with the QI pass rate.

## Discussion and conclusions

In the present study, we found that the median summary OA-QI pass rate was 47%, which is higher than the study by Østerås et al., which recruited participants from a population based survey and also included persons with hand OA [18]. Also, we found no difference between pass rates for the non-pharmacological and pharmacological treatments, which is contrary to Østerås et al. Nevertheless, the fact that the referral for weight reduction had the lowest pass rate and that having received information about the importance of physical activity and exercise had the highest pass rate is similar to the findings by Østerås et al. [18]. The fact that very few of those who self reported being overweight had not been referred to weight reduction might simply reflect that there is currently no community based service for moderately overweight people in Norway, but also that this question is prone to recall bias. Similarly, the high pass rate for physical activity and exercise might reflect that that the importance of exercise is emphasized by health authorities and in mass media, that mean people will remember and respond to advice in this area but may be not for weight loss. Despite the relatively low pass rates on OA-QI, the OA patients were quite satisfied with the treatment they had received. We also found a significant bivariate association between OA-QI pass rate and satisfaction. However, this association may be confounded by other variables, for example number of GP consultations, and should be interpreted with caution.

The present study has several limitations. Firstly, we cannot know that the patients that returned the questionnaire were representative of the OA patients normally treated by the GPs. The study sample may be have been subject to selection bias related to the GPs’ recruiting participants. Although the GP offices were monitored with telephone follow-ups and personal visits, we don’t know exactly how many eligible patients who attended the clinics. Secondly, the response rate was relatively low, though still comparable to a similar study [19]. The low response rate, as well as the possible selection bias related to recruitment, limited the generalizability of the results since we cannot be certain that the respondents were not systematically different from those not invited and non-responding. Also the fact that the respondents reported a relatively low BMI and a high education level suggests recruitment bias in the present study. Thirdly, we did not standardize the procedure for completing and returning of the questionnaires, and the fact that some patients completed the questionnaire at the GP offices might have biased the responses toward higher pass rates. Because patient self-reported data could be biased due to recall bias, as well as patient factors such as illness perception and treatment beliefs, this may also have affected the responses. For example, there is evidence that overweight and obese people tend to underreport their BMI, whereas very few, if any over report their BMI. Thus, we can assume that those who reported overweight in the present study actually are overweight, whereas there might be a proportion of ‘false negatives’ that have not responded to the question about advice and referral for overweight.

The OA-QI questionnaire has not been validated against medical records. It has been suggested that self-reported assessment tools tend to score the same or higher than medical records and QI assessment made by care providers [20]. If this overestimation of the care given has influenced the results in this study, it may have caused the pass rates from OA-QI to be overrated. Finally, we assumed the 17 items to be of equal importance when we calculated the overall pass rate. Since most evidence based clinical guidelines employ a system with grading the strength of recommendations, this may also be reflected by giving the items different weight in the overall pass rate. Also the patient perspective should be included, when considering a weighted overall pass rate.

One strength of the study was the use of the OA-QI questionnaire. Assessing care directly from the patients, either via interviews or using patient reported QIs, makes it possible to assess the care received and/or perceived, although this is not necessarily the same as what the GP or others have been given and what is stated in the patient records. We think that assessing what the patient remembers or perceived of the care given is vital due to the compliance of the care. Another strength of the study is that the inclusion of patients was made directly by the GPs, as this method of inclusion yielded a very high compliance rate regarding fulfilment of the inclusion criteria since none of the returned questionnaires were regarded as being outside the inclusion criteria. A third strength of the study is the assessment of the patients’ comorbidities. Although self-reported, this provides a better understanding of the complexity of the patients and everyday choices the GPs have to make regarding what care to prescribe. Furthermore, the comorbidities may also help explain some of the variations in the fulfilment of different QIs.

Others have assessed the quality of the OA treatment using different QI sets than what we used in the present study. For example, Ascari et al. [21] reviewed the use of the ACOVE QIs in 17 studies and found that the interquartile range score of 29-41% for OA was the lowest score among the diseases reviewed [22], while Li et al. [19] reported the pass rate on four QIs to be 22%. However, comparing QI pass rates should be done with caution because the study samples, settings and methods may differ. It is also worth mentioning that comorbidity is common among persons with OA and may affect the QI pass rates due to the fact that patients may not remember for which disease they achieved information, and there may be conflicts between recommendations for comorbidities and OA care, thereby making it difficult for the GPs to choose between different recommendations [22]. In this study, 72% reported having at least one chronic disease other than OA, though the rates of comorbidities in the other studies assessing QIs were not reported.

In our study the summary QI pass rate was 47%, which is a higher pass rate than what others have reported, and which may be due to the characteristics of the participants in the present study. Most studies assessing OA patients report the patients as having a higher BMI and lower education than the average population, and in our study sample the average BMI was 27.4. Although a BMI>25 is regarded as being overweight, it is the same as the average BMI for the adult population in Norway [23]. Our respondents have a higher education level than average, with 20% reporting having a university education, while the average for Nord-Trøndelag County is 3.9% [24]. Thus, the differences in characteristics between the respondents in the present study and respondents in other studies may help explain some of the differences in pass rates.

Even if our study sample might not be representative for all OA patients in Norway, and may be overestimating the quality of OA care, the overall pass rate was only 47%, leaving substantial room for improvement. Guidelines for OA care have been available for more than a decade, yet implementation in daily practice is revealed as lacking in more than 50% of the QIs assessed, including in both pharmacological and non-pharmacological OA care. Future research and efforts for improving OA care should be directed towards the implementation of existing guidelines.

## Additional files

Additional file 1:
**HOOS HIP SURVEY.**
Additional file 2:
**OsteoArthritis Quality Indicator questionnaire (OA-QI).**
