# Supplementary material for: Quality of hip and knee osteoarthritis management in primary health care in a Norwegian county: a cross-sectional survey
Source: BMC Health Serv Res. 2014 Nov 25;14:598. doi: 10.1186/s12913-014-0598-x (PMC4252009; doi:10.1186/s12913-014-0598-x)
Supplement: Additional file 1: — HOOS HIP SURVEY. [file 12913_2014_598_MOESM1_ESM.pdf]

HOOS HIP SURVEY

Today's date: _____/______/______ Date of birth: _____/______/________

Name: ____

INSTRUCTIONS: This survey asks for your view about your hip. This information will help us keep track of how you feel about your hip and how well you are able to do your usual activities.
Answer every question by ticking the appropriate box, only one box for each question. If you are uncertain about how to answer a question, please give the best answer you can.

Symptoms

These questions should be answered thinking of your hip symptoms and difficulties during the **last week**.

S1. Do you feel grinding, hear clicking or any other type of noise from your hip?

Never	Rarely	Sometimes	Often	Always
☐	☐	☐	☐	☐

S2. Difficulties spreading legs wide apart

None	Mild	Moderate	Severe	Extreme
☐	☐	☐	☐	☐

S3. Difficulties to stride out when walking

None	Mild	Moderate	Severe	Extreme
☐	☐	☐	☐	☐

Stiffness

The following questions concern the amount of joint stiffness you have experienced during the **last week** in your hip. Stiffness is a sensation of restriction or slowness in the ease with which you move your hip joint.

S4. How severe is your hip joint stiffness after first wakening in the morning?

None	Mild	Moderate	Severe	Extreme
☐	☐	☐	☐	☐

S5. How severe is your hip stiffness after sitting, lying or resting **later in the day**?

None	Mild	Moderate	Severe	Extreme
☐	☐	☐	☐	☐

Pain

P1. How often is your hip painful?

Never	Monthly	Weekly	Daily	Always
☐	☐	☐	☐	☐

What amount of hip pain have you experienced the **last week** during the following activities?

P2. Straightening your hip fully

None	Mild	Moderate	Severe	Extreme
☐	☐	☐	☐	☐

What amount of hip pain have you experienced the **last week** during the following activities?

P3. Bending your hip fully

None	Mild	Moderate	Severe	Extreme
☐	☐	☐	☐	☐

P4. Walking on a flat surface

None	Mild	Moderate	Severe	Extreme
☐	☐	☐	☐	☐

P5. Going up or down stairs

None	Mild	Moderate	Severe	Extreme
☐	☐	☐	☐	☐

P6. At night while in bed

None	Mild	Moderate	Severe	Extreme
☐	☐	☐	☐	☐

P7. Sitting or lying

None	Mild	Moderate	Severe	Extreme
☐	☐	☐	☐	☐

P8. Standing upright

None	Mild	Moderate	Severe	Extreme
☐	☐	☐	☐	☐

P9. Walking on a hard surface (asphalt, concrete, etc.)

None	Mild	Moderate	Severe	Extreme
☐	☐	☐	☐	☐

P10. Walking on an uneven surface

None	Mild	Moderate	Severe	Extreme
☐	☐	☐	☐	☐

Function, daily living

The following questions concern your physical function. By this we mean your ability to move around and to look after yourself. For each of the following activities please indicate the degree of difficulty you have experienced in the **last week** due to your hip.

A1. Descending stairs

None	Mild	Moderate	Severe	Extreme
☐	☐	☐	☐	☐

A2. Ascending stairs

None	Mild	Moderate	Severe	Extreme
☐	☐	☐	☐	☐

A3. Rising from sitting

None	Mild	Moderate	Severe	Extreme
☐	☐	☐	☐	☐

A4. Standing

None	Mild	Moderate	Severe	Extreme
☐	☐	☐	☐	☐

For each of the following activities please indicate the degree of difficulty you have experienced in the **last week** due to your hip.

A5. Bending to the floor/pick up an object

None	Mild	Moderate	Severe	Extreme
☐	☐	☐	☐	☐

A6. Walking on a flat surface

None	Mild	Moderate	Severe	Extreme
☐	☐	☐	☐	☐

A7. Getting in/out of car

None	Mild	Moderate	Severe	Extreme
☐	☐	☐	☐	☐

A8. Going shopping

None	Mild	Moderate	Severe	Extreme
☐	☐	☐	☐	☐

A9. Putting on socks/stockings

None	Mild	Moderate	Severe	Extreme
☐	☐	☐	☐	☐

A10. Rising from bed

None	Mild	Moderate	Severe	Extreme
☐	☐	☐	☐	☐

A11. Taking off socks/stockings

None	Mild	Moderate	Severe	Extreme
☐	☐	☐	☐	☐

A12. Lying in bed (turning over, maintaining hip position)

None	Mild	Moderate	Severe	Extreme
☐	☐	☐	☐	☐

A13. Getting in/out of bath

None	Mild	Moderate	Severe	Extreme
☐	☐	☐	☐	☐

A14. Sitting

None	Mild	Moderate	Severe	Extreme
☐	☐	☐	☐	☐

A15. Getting on/off toilet

None	Mild	Moderate	Severe	Extreme
☐	☐	☐	☐	☐

A16. Heavy domestic duties (moving heavy boxes, scrubbing floors, etc)

None	Mild	Moderate	Severe	Extreme
☐	☐	☐	☐	☐

A17. Light domestic duties (cooking, dusting, etc)

None	Mild	Moderate	Severe	Extreme
☐	☐	☐	☐	☐

Function, sports and recreational activities

The following questions concern your physical function when being active on a higher level. The questions should be answered thinking of what degree of difficulty you have experienced during the **last week** due to your hip.

SP1. Squatting

None	Mild	Moderate	Severe	Extreme
☐	☐	☐	☐	☐

SP2. Running

None	Mild	Moderate	Severe	Extreme
☐	☐	☐	☐	☐

SP3. Twisting/pivoting on loaded leg

None	Mild	Moderate	Severe	Extreme
☐	☐	☐	☐	☐

SP4. Walking on uneven surface

None	Mild	Moderate	Severe	Extreme
☐	☐	☐	☐	☐

Quality of Life

Q1. How often are you aware of your hip problem?

Never	Monthly	Weekly	Daily	Constantly
☐	☐	☐	☐	☐

Q2. Have you modified your life style to avoid activities potentially damaging to your hip?

Not at all	Mildly	Moderately	Severely	Totally
☐	☐	☐	☐	☐

Q3. How much are you troubled with lack of confidence in your hip?

Not at all	Mildly	Moderately	Severely	Extremely
☐	☐	☐	☐	☐

Q4. In general, how much difficulty do you have with your hip?

None	Mild	Moderate	Severe	Extreme
☐	☐	☐	☐	☐

Thank you very much for completing all the questions in this questionnaire.
