# Supplementary material for: Quality of hip and knee osteoarthritis management in primary health care in a Norwegian county: a cross-sectional survey
Source: BMC Health Serv Res. 2014 Nov 25;14:598. doi: 10.1186/s12913-014-0598-x (PMC4252009; doi:10.1186/s12913-014-0598-x)
Supplement: Additional file 2: — OsteoArthritis Quality Indicator questionnaire (OA-QI). [file 12913_2014_598_MOESM2_ESM.pdf]

Questions on the treatment of your osteoarthritis

There are several different treatment alternatives for osteoarthritis. We would like to know what treatment, information or advice that you have been given for your osteoarthritis. For each question, please cross off one of the boxes provided.

		Yes	No	Don't remember
1	Have you been given information about how the disease usually develops over time?	☐	☐	☐
2	Have you been given information about different treatment alternatives?	☐	☐	☐
3	Have you been given information about how you can live with the disease?	☐	☐	☐
4	Have you been given information about how you can change your lifestyle?	☐	☐	☐
5	Have you been given information about the importance of physical activity and exercise?	☐	☐	☐
6	Have you been referred to someone who can advise you about physical activity and exercise? (e.g. a physiotherapist)	☐	☐	☐

		Yes	No	Not overweight
7	If you are overweight, have you been advised to lose weight?	☐	☐	☐
8	If you are overweight, have you been referred to someone who can help you to lose weight?	☐	☐	☐

		Yes	No	No such problems
9	If you have had problems related to daily activities, have these problems been assessed by health personnel in the past year?	☐	☐	☐
10	If you have problems with walking, has your need for a walking aid been assessed? (e.g. stick, crutch or walker)	☐	☐	☐
11	If you have problems related to other daily activities, has your need for different appliances and aids been assessed? (e.g. splints, assistive technology for cooking or personal hygiene, a special chair)	☐	☐	☐

		Yes	No	No pain/ discomfort
12	If you have pain, has it been assessed in the past year?	☐	☐	☐
13	If you have pain, was paracetamol the first medicine that was recommended for your osteoarthritic pain?	☐	☐	☐
14	If you have prolonged severe pain, which is not relieved sufficiently by paracetamol, have you been offered stronger pain killers? (e.g. Co-proxamol, Co-dydramol, Tramadol, Co-codamol, Dihydrocodeine, Codeine)	☐	☐	☐
15	If you are taking anti-inflammatory drugs, have you been given information about the effects and possible side-effects of this medicine? (e.g. Ibuprofen, Nurofen, Brufen, Diclofenac, Voltarol, Naproxen, Naprosyn, Celebrex)	☐	☐	☐
16	If you have experienced an acute deterioration of your symptoms, has a corticosteroid injection been considered?	☐	☐	☐

		Yes	No	Not severely troubled
17	If you are severely troubled by your osteoarthritis, and exercise and medicine do not help, have you been referred and assessed for an operation (e.g. joint replacement)?	☐	☐	☐
